# EP-YOLO: An Enhanced Lightweight Model for Micro-Pest Detection in Agricultural Light-Trap Environments

**DOI:** 10.3390/s26092607

**Published:** 2026-04-23

**Authors:** Yuyang Tang, Jiaxuan Wang, Wenxi Sheng, Jilong Bian

**Affiliations:** 1 Aulin College, Northeast Forestry University, Harbin 150040, China; 2023214664@nefu.edu.cn (Y.T.); 2023224713@nefu.edu.cn (J.W.); shengwenxi@nefu.edu.cn (W.S.); 2 College of Computer and Control Engineering, Northeast Forestry University, Harbin 150040, China

**Keywords:** pest detection, object detection, YOLOv8, lightweight networks, attention mechanism

## Abstract

As food security gains increasing attention, automated pest monitoring is crucial for agricultural early warning systems. However, in practical light-trap capturing sensors, the extremely small scale of pests and complex background interference, such as unexpected reflection and occlusions, severely undermine the performance of existing models, resulting in frequent missed and false detections. To deal with these challenges, this study proposes EP-YOLO, an enhanced lightweight detection architecture based on YOLOv8n. Specifically, to retain the spatial pixels of micro-targets during downsampling and isolate pest features while eliminating background noise without compromising channel information, the Spatial-to-Depth Convolution (SPD) module and the Efficient Multi-Scale Attention (EMA) module are introduced. We evaluate our model through experiments on Pest24, a dataset consisting of 24 tiny pest categories. The results demonstrate that EP-YOLO achieves a mAP@50 and mAP@50:95 of 70.5% and 47.3%, respectively, improving upon the baseline by 1.1% and 1.9%. Furthermore, EP-YOLO achieves a significant improvement in detecting certain extremely small pests. For example, *Rice planthopper* and *Plutella xylostella* show improvements of 8.4% and 3.1%, respectively, compared to the baseline. In conclusion, the physical limitations of detecting tiny pests are successfully overcome by EP-YOLO, providing a robust and deployable design for real-time agricultural monitoring systems.

## 1. Introduction

Global agricultural development and food security are continuously under the threats of crop pests [1,2]. To minimize the use of chemical pesticides, accurate and timely monitoring of pest population is essential [3,4]. Traditionally, pest identification heavily relied on manual detection approaches, which is laborious, error-prone and inefficient. With the rapid development of smart agriculture and Internet of Things (IoT) technologies, intelligent monitoring systems equipped with visual sensors have emerged as the popular solution for real-time pest detection in the field, such as automated light-trap devices and sticky board trap [5,6]. Although the potential of these advanced technologies is outstanding, images captured by them pose significant problems for existing computer vision models. First, the target pests in the images are often at an extremely small scale, and they are prone to distribute in an irregular pattern. For example, overlapping and dense packing are common in a single picture. Second, unexpected and complex background noise make detection even harder, such as unpredictable illumination changes made by the light-trap devices [7]. In the broader scope of agricultural computer vision, researchers also confront severe target occlusions [8,9], the extraction of subtle visual patterns in plant diseases [10,11], and the necessity of multi-sensor data fusion to overcome single-modal limitations [10,12]. While these challenges are prominent across general agriculture, the light-trap environment focused on in this study presents a uniquely extreme combination of tiny target scales and intense optical interference, which requires a specialized architecture.

In the early stages of automated pest monitoring, researchers mainly relied on manual feature extraction and traditional machine learning. For instance, Ebrahimi et al. [13] utilized Support Vector Machines (SVM) based on explicitly extracted shape and color features for pest classification. Similarly, Deng et al. [14] explored bio-inspired methods combined with local features for pest recognition. These traditional methods achieved certain successes under controlled conditions. However, their performance is significantly undermined by the complex backgrounds and extreme scale variations typical of wild field images.

To overcome these limitations, deep learning has become a prevalent mechanism in agricultural detection. Due to the outstanding accuracy in generating region proposals and locating targets, two-stage object detectors such as Faster R-CNN received huge attention [15]. For example, Wang et al. [16] proposed an S-RPN module to address sampling imbalance in small crop pest detection. This modification significantly improved the recall rate of tiny insects. Similarly, Li et al. [17] developed a deep learning framework for detecting tiny pests like whiteflies on sticky traps. Moreover, their team have also introduced Faster-PestNet [18] to enhance crop pest classification performance.

Despite their excellent detection performance, the two-stage architectures suffer from a critical bottleneck. They rely on dense region proposals and complex classification heads, which brings in massive parameter counts and high computational overhead [19]. revMeanwhile, agricultural IoT visual sensor devices are strictly constrained by low power budgets, limited memory storage, and the need for real-time processing. Consequently, it is inappropriate to deploy such heavy and computationally costly models on these edge devices [20].

Single-stage detectors such as the YOLO series have been widely used to meet the real-time processing requirements of agricultural edge sensor devices. In recent researches, modifying YOLO architectures to tackle the challenge of detecting tiny pests has become a focal point [21,22]. For instance, Guo et al. [23] developed the YOLO-SIP detector by adding a new prediction module to the head to detect flying vegetable pests. Similarly, Chen et al. [24] enhanced YOLOv8l by integrating a P2 (i.e., a high-resolution detection layer with a stride of 4) micro-target detection head and an Adaptively Spatial Feature Fusion module in the neck to fuse multi-scale features.

Tian et al. [25] proposed MD-YOLO, which extends the feature pyramid to fuse shallower, high-resolution feature maps alongside an extra prediction head. Furthermore, Tang et al. [26] improved Pest-YOLO by employing cross-stage feature fusion (CSFF) and a transformer encoder to capture global features for pests in multiple classes. These backend enhancements, such as introducing P2 heads or fusing shallower feature maps, succeed in preserving high-resolution details. revMoreover, with the rapid iteration of the YOLO series, the latest architectures such as YOLOv10 and YOLOv11 have also been employed to further push the boundaries of inference efficiency in agricultural applications [27,28].

However, they share the following fundamental architectural limitation: they still operate on feature maps that have undergone initial strided downsampling in the early stages of the standard backbones. These feature maps are processed by the conventional strided convolutions. Therefore, the modifications from the backend are forced to construct localization information from already degraded features. This acts as a sub-optimal remedy rather than preventing the spatial information loss at the source.

On the other hand, complex background noise remains a critical challenge for agricultural visual sensors. It is difficult to suppress noise without adding extra parameters. A common approach to these issues is to integrate various attention modules into the network. For example, Zhang et al. [29] proposed AgriPest-YOLO, which designed and incorporated a Coordination and Local Attention module to mitigate background noise. Wang et al. [30] introduced the Convolutional Block Attention Module (CBAM) into Insect-YOLO to enhance the feature extraction capabilities for tiny pests. Similarly, Xiang et al. [31] designed the Yolo-Pest algorithm featuring a CAC3 module that leverages coordinate attention to focus on pest targets. Moreover, Cen et al. [32] developed YOLO-LCE, applying Efficient Channel Attention (ECA) as well as a redesigned lightweight bottleneck to reduce parameters. Integrating attention modules successfully enhances the model’s ability to suppress background interference and facilitate the extraction of features of tiny pests. However, these modules still possess notable drawbacks. For instance, mechanisms like CBAM utilize large convolution kernels, which exert an extra burden on the computational resources. Furthermore, modules like ECA only employ channel attention, making it difficult to capture cross-dimensional features. In addition to CNN-based attention, the recent literature highlights the trend of utilizing Transformer-based self-attention mechanisms to capture global dependencies in agricultural scenes [33,34]. However, the massive computational complexity inherently associated with standard self-attention often leads to a huge parameter surge. This severely contradicts the low-power deployment constraints of agricultural IoT edge sensors.

The current research gaps in micro-pest detection for light-trap environments can be summarized into the following three main aspects:

- Conventional strided convolutions inevitably cause premature loss of fine-grained spatial information.
- Existing attention mechanisms either introduce excessive computational overhead or fail to efficiently capture cross-dimensional spatial and channel dependencies to suppress severe background noise.
- Due to the strict parameter constraints required by agricultural IoT edge devices, there is a lack of algorithms that can reach an optimal balance between high-precision micro-pest detection.

Therefore, the primary research questions of this study are as follows: (1) how to prevent the premature loss of fine-grained spatial information for extremely small pests at the architectural source, and (2) how to strategically integrate cross-dimensional attention mechanisms to filter out severe background noise without compromising the low-power deployment feasibility of the overall model.

To solve the defects mentioned above, a lightweight pest detection model named EP-YOLO is proposed in this study. Built upon the YOLOv8n baseline, EP-YOLO integrates the Space-to-Depth Convolution (SPDConv) and the Efficient Multi-Scale Attention (EMA) module. Specifically, parts of the traditional strided convolutions in the backbone are replaced by SPDConv. This module enables lossless feature downsampling, significantly mitigating the premature loss of fine-grained pixel details for extremely tiny pests. Moreover, we integrate the EMA module in order to suppress complex background noise. This lightweight attention mechanism is able to capture cross-dimensional spatial and channel dependencies with minimal parameter increment. The main contributions of this paper are outlined as follows:

- A lightweight single-stage detector EP-YOLO is proposed in this study, which is specifically tailored for agricultural visual sensor devices. Experimental results demonstrates that EP-YOLO achieves superior detection capability on complex light-trap images.
- We identify the inherent limitations of conventional strided convolutions in handling low-resolution, tiny targets. As a result, we introduced SPD to capture lossless detailed features during early-stage downsampling.
- The EMA modules are integrated into the network architecture, providing a highly efficient cross-dimensional attention mechanism that effectively suppresses background noise.
- We verified EP-YOLO through comprehensive experiments on a highly challenging dataset Pest24. The model significantly boosts the detection accuracy of the most demanding pest categories while maintaining a small parameter scale of 3.02 M, providing a highly practical and efficient solution for agricultural IoT deployment.

## 2. Materials and Methods

### 2.1. Dataset Introduction

To evaluate our model, we selected Pest24 as the benchmark dataset. Pest24 is a public dataset for multi-target pest detection tasks. The images are captured by automated pest trapping devices in real field environments, encompassing 24 typical agricultural pests and 25,378 annotated images in total [7]. The original resolution of images is 2095 × 1944. However, several features of the dataset pose significant challenges for precise detection.

**Figure 1:**
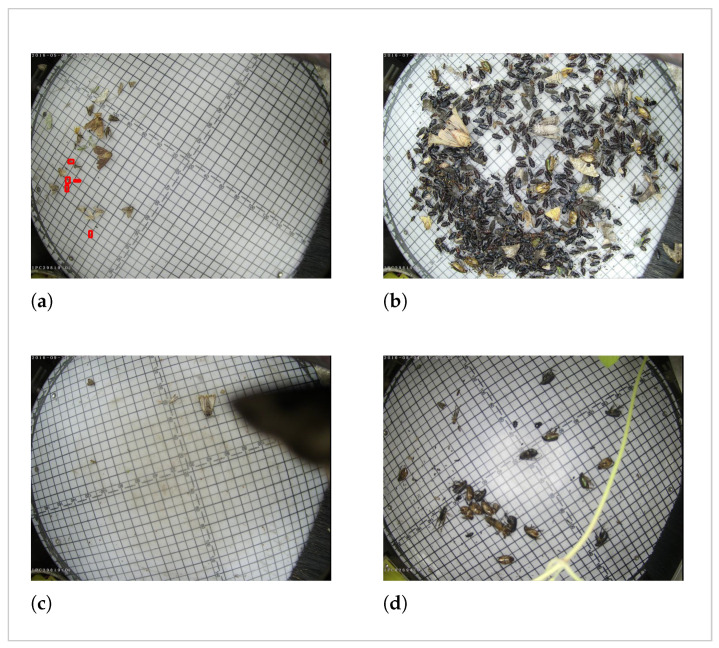
Visual examples of the main challenges in the Pest24 dataset: (**a**) Extremely small targets, the red boxes indicate the locations of extremely small pests. (**b**) Dense distribution and severe overlap/Scale variation; (**c**,**d**) Unexpected objects occlusions.

First, the dataset presents severe instance-level and distribution challenges as follows:

- Extremely small targets and drastic scale variations. As shown in Figure 1a, the dataset contains pests in a significantly small scale. Meanwhile, in Figure 1b, a large-scale variation between different categories can be observed. [7,29].
- Dense distribution and severe overlap. The pests in the image are densely distributed, which results in frequent adhesions and overlapping instances as displayed in Figure 1b. Among all the images, there exists at most 214 targets in one picture, and up to 4657 of them feature non-negligible object adhesions [7].
- Long-tailed data distribution. As shown in Table 1, the distribution within the dataset is highly imbalanced. For example, the most frequent category (*Anomala corpulenta*) contains 53,347 instances, whereas the least frequent (*Holotrichia oblita*) has only 108 instances [7].

Second, the dataset introduces complex environmental and background challenges, as follows:

- Illumination variations and reflection spots. The flashlight mounted on the trapping devices create unexpected reflection spots and lighting inconsistencies [29].
- Shadows and occlusion. Approximately 600 images contain unexpected shadows and physical occlusions, which introduce irregular and unpredictable visual noise [7]. Figure 1c,d demonstrate two examples of unexpected occlusions that exist in our dataset.
- Non-targeted pest interference. Aside from the annotated positive targets, the images contain plenty of unannotated, non-targeted insects that share a high similarity with the target pests [7].

In conclusion, these challenging features make Pest24 a highly demanding dataset for training robust object detectors.

### 2.2. The Proposed Structure of EP-YOLO

#### 2.2.1. Baseline Model and Its Limitations

As one of the most advanced anchor-free, single-stage object detectors currently available, YOLOv8 has achieved significant breakthroughs in comprehensive performance. Its network architecture primarily consists of a modified CSPDarknet-based backbone [35] for feature extraction, a PANet for multi-scale feature aggregation, and a decoupled head. In this study, considering that automated agricultural pest trapping devices are constrained by the computational power [3], we selected YOLOv8n, the most lightweight version in the YOLOv8 series, as our baseline model. YOLOv8n guarantees a highly efficient inference speed while maintaining an extremely low number of parameters and computational complexity, making it an ideal baseline architecture for real-time pest monitoring in the field.

However, although YOLOv8n performs excellently in general object detection tasks, it exposes two critical performance bottlenecks when applied to specific agricultural datasets like Pest24, as follows: extremely small targets and complex backgrounds.

First, because of the tiny scale of those target pests, the network is prone to lose fine-grained features during the downsampling process. The backbone of YOLOv8n heavily relies on 3×3 convolutions with a stride of 2 to reduce the feature maps’ dimensionality and expand the receptive field. For the numerous micro-scale pests in Pest24, continuous strided convolutions inevitably destroy their already scarce pixel information. As the network deepens, the features of these extremely small targets degrade or even completely disappear, ultimately leading to severe false negatives [36,37].

Second, the network is sensitive to complex background noise during the feature fusion stage. When performing multi-scale feature concatenation, the original PANet structure lacks a mechanism to effectively allocate weight across spatial and channel dimensions [38]. In real-world field scenarios, light-trap capture boards often contain unpredictable noise interference, such as local reflections and shadows caused by equipment flashlights, and unexpected occlusions. Unable to filter the background, the backbone encodes these noises along with the features of pests. When these noisy multi-scale features are fed into the original PANet, the background interference is severely fused and amplified with the target’s features. As a result, a great deal of non-target objects are misclassified as targets, resulting in a high false positive rate.

In summary, to overcome the inherent defects of the baseline model in retaining micro-features and suppressing background noise, we performed a targeted architectural reconstruction based on YOLOv8n and proposed the EP-YOLO model.

#### 2.2.2. Architecture of the Proposed EP-YOLO

**Figure 2:**
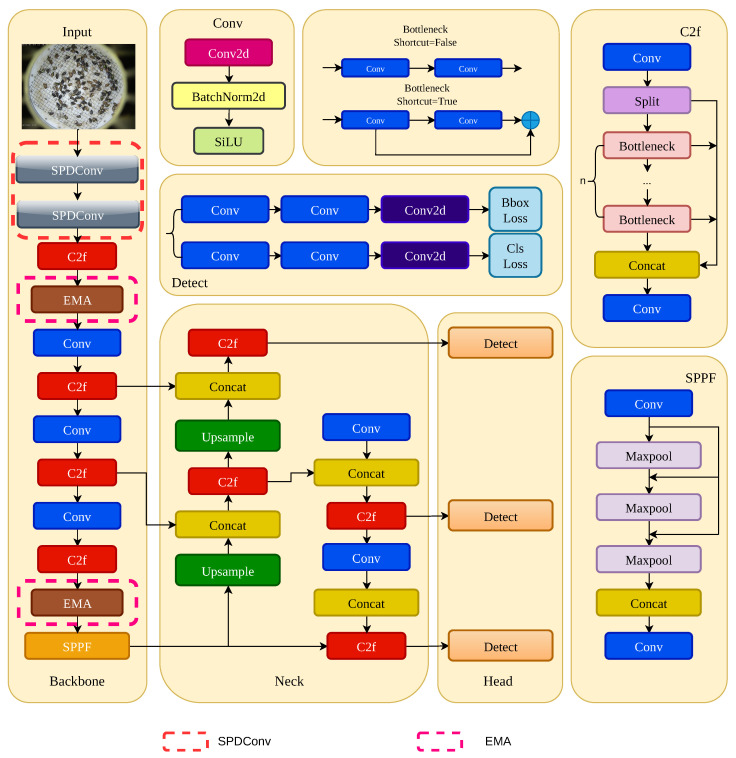
The overall architecture of the proposed EP-YOLO. (The arrows indicate the flow of feature maps, and ‘…’ denotes the repetition of bottleneck blocks).

To address the limitations of the baseline model in complex agricultural scenarios, this paper proposes EP-YOLO (EMA-Pixel YOLO), a novel network specifically optimized for micro-pest detection. The overall network of EP-YOLO is illustrated in Figure 2. After the raw image captured by the trapping device is fed into the network, it first enters the reconstructed Stem layer, which serves as the primary visual processing stage responsible for initial feature extraction and spatial dimensionality reduction. Unlike the original YOLOv8n, which employs conventional strided convolutions, we introduce SPD-Conv at the very front end of the network [39]. This improvement effectively prevents the loss of pixel information during the early downsampling process, ensuring that the high-resolution, fine-grained features of extremely small pests are fully preserved and transmitted to the subsequent backbone [40].

As the network layers deepen, the receptive field of the feature maps expands rapidly, and high-dimensional semantic information begins to aggregate. However, strong background noise, such as reflections, is encoded simultaneously. To mitigate this, before the features enter the Neck for fusion, we embed the Efficient Multi-Scale Attention (EMA) modules within the backbone. The EMA module re-weights the feature channels through cross-spatial information aggregation. During this process, features highly correlated with the pests themselves are enhanced, while redundant features associated with background noise are effectively suppressed. This ensures that the features fed into subsequent layers possess high purity and discriminative power.

Finally, the multi-scale feature maps “purified” by the EMA module enter the FPN-PAN structure for top-down and bottom-up bidirectional fusion. The fused features across three scales are then passed into the Decoupled Head to independently perform classification and bounding box regression tasks. Benefiting from the physical preservation of micro pixels by the front-end SPD module and the precise filtering of complex backgrounds by the EMA modules, EP-YOLO significantly improves the detection accuracy of micro-pests in the final prediction stage.

### 2.3. Spatial-to-Depth Convolution

**Figure 3:**
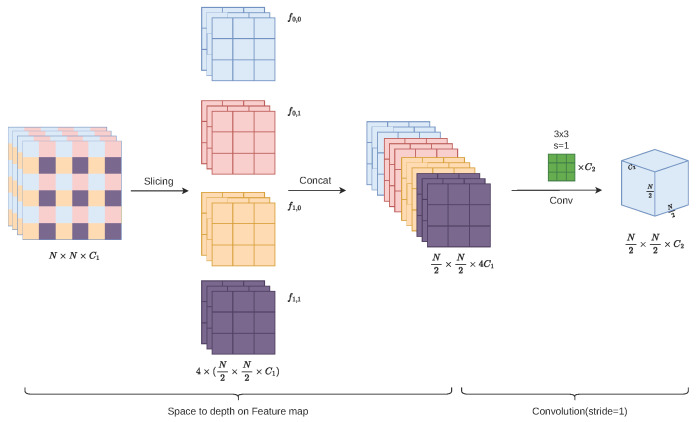
The detailed structure of the SPD-Conv module.

As mentioned in Section 2.2.1, traditional strided convolutions cause loss of tiny pest features. To deal with this issue, we introduce the Space-to-Depth module in the early stage of the EP-YOLO backbone. SPDConv works as a front-end feature preserver; it is capable of minimizing the loss of information during the downsampling process. The comprehensive network structure is shown in Figure 3.

The SPD-Conv is composed of two main procedures, namely, Space-to-Depth slicing and the Non-strided Convolution. First, when the module is fed with a feature map *X* with dimensions $N\times N\times C_{1}$, the Space-to-Depth layer starts to slice the spatial dimension alternately according to a given downsampling factor scale. This process can be expressed by Equation (1) as follows:

(1) $$f_{x,y}=X\left[x:N:scale,y:N:scale\right],\;x,y\in \left\{0,1,\ldots ,scale-1\right\}$$

where *X* denotes the original input, and $f_{x,y}$ denotes the sequence of sub-feature maps after slicing.

In our scenario, the scale is set to 2. Therefore, the Space-to-Depth layer slices the feature map and extracts the pixels at an interval of 2 in both the width and height directions (the network is collecting pixels starting from coordinates (0,0), (0,1), (1,0), and (1,1), respectively). As a result, four independent sub-feature maps with dimensions $\frac{N}{2}\times \frac{N}{2}\times C_{1}$ are extracted, corresponding to the sequences $f_{0,0}$, $f_{0,1}$, $f_{1,0}$, $f_{1,1}$ shown in Figure 3.

After the collection of these four sub-feature maps, the network concatenates them along the channel dimension, which can be expressed by Equation (2) as follows:

(2) $$X_{concat}=Concat\left(\left[f_{0,0},f_{0,1},f_{1,0},f_{1,1}\right],dim=C\right)$$

where Concat denotes the concatenation along the channel dimension *C*.

Due to this operation, the original spatial pixel information is fully retained, constructing an intermediate tensor $X_{concat}$. $X_{concat}$ has an expanded channel dimensions of $\frac{N}{2}\times \frac{N}{2}\times 4C_{1}$.

Finally, the network leverages a 3×3 convolution with a stride of 1 on the intermediate tensor $X_{concat}$ to further integrate cross-channel semantics and restrain the model parameter. The process is expressed by Equation (3) as follows:

(3) $$X_{out}=Conv_{3\times 3}\left(X_{concat},stride=1\right)$$

where $X_{out}$ is the final output feature map of the SPD-Conv module.

Through this final operation, a feature map with dimensions of $\frac{N}{2}\times \frac{N}{2}\times C_{2}$ is generated. This feature map avoids the micro-feature loss caused by traditional strided convolutions, achieving an effective spatial downsampling.

The detection challenges of the Pest24 dataset are effectively addressed by this feature rearrangement mechanism. Traditionally, strided convolutions scan the feature map with a stride of 2, in which only 1 out of every 4 adjacent pixels in the input is preserved. While in regular large-scale target detection tasks, this information discarding pattern is negligible, it is catastrophic for tiny pests. In contrast, the SPD-Conv losslessly captures all pixels within the local receptive field due to the special operation of transferring information from the spatial dimension to the channel dimension. This design ensures that the features of micro-pests are completely transmitted after the early downsampling operations. Therefore, the phenomenon of missed detections at the source of the backbone is mitigated.

### 2.4. Efficient Multi-Scale Attention

**Figure 4:**
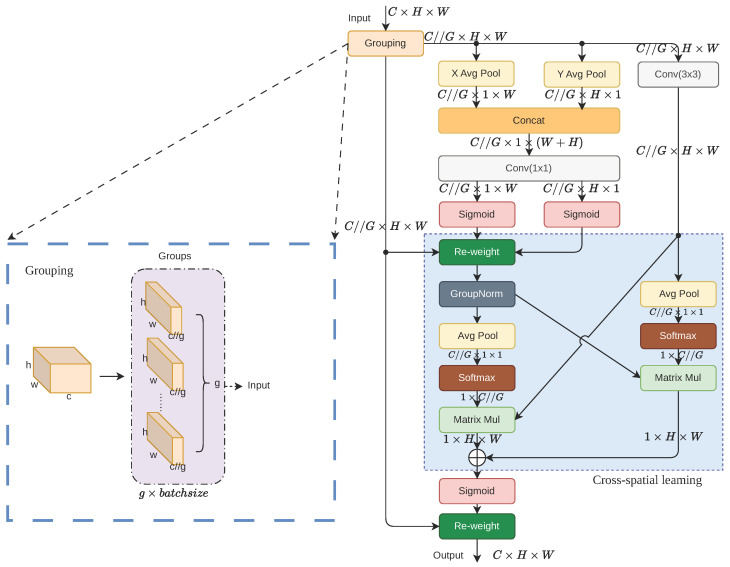
The internal working mechanism of the Efficient Multi-Scale Attention (EMA) module. (Solid arrows indicate data flow, ‘…’ represents repeated modules, and colors distinguish functional blocks).

To address the issue pointed out in Section 2.2.1 that the baseline model is highly sensitive to complex background noise during the feature fusion stage, we introduce the Efficient Multi-Scale Attention (EMA) module into the EP-YOLO backbone. Specifically, EMA is embedded into the shallow stage of the network, as well as the deep stage. The design inspiration of EMA stems from the Coordinate Attention mechanism [41]. Both are dedicated to encoding precise 2D spatial position information into channel attention. However, Coordinate Attention overly relies on channel dimensionality reduction during this process, which inevitably leads to partial feature loss. To resolve this flaw, EMA adopts a design of grouping and multi-scale parallel subnetworks, extracting attention weights under the premise of completely avoiding the compression of channel thickness. Its internal working mechanism is illustrated in Figure 4.

When a feature map $X\in R^{C\times H\times W}$ carrying high-dimensional semantic information is input into the EMA module, the network first uniformly slices it into *G* sub-feature groups along the channel dimension. The core formula is expressed as follows:

(4) $$X=\left[X_{0},X_{1},\ldots ,X_{G-1}\right],\;X_{i}\in R^{C//G\times H\times W}$$

where *C*, *H*, and *W* represent the total number of channels, height, and width of the input feature map, respectively, and *G* is the preset total number of groups. Inside each feature group, the data flow is distributed to two heterogeneous parallel feature extraction branches at the same time. In the 1×1 branch, the network executes 1D global pooling, X Avg Pool and Y Avg Pool, along the horizontal and vertical directions, respectively. The working mechanism of this process can be expressed by the following formulas:

(5) $$z_{c}^{H}\left(H\right)=\frac{1}{W}\sum_{i=1}^{W}x_{c}\left(H,i\right),\;z_{c}^{W}\left(W\right)=\frac{1}{H}\sum_{j=1}^{H}x_{c}\left(j,W\right)$$

Here, $x_{c}$ represents the input feature component on the *c*-th channel, and $z_{c}^{H}$ and $z_{c}^{W}$ characterize the 1D position information encoding the global receptive field in the height and width directions, respectively. Subsequently, these two sets of features are concatenated and fed into a 1×1 convolution layer to obtain inter-channel feature information. Meanwhile, the parallel 3×3 branch directly utilizes a 3×3 convolution kernel to extract local multi-scale spatial details. To break the state of feature isolation between the two branches, the network designs a Cross-spatial learning mechanism at the end. This mechanism utilizes 2D global pooling and a Softmax function to generate a channel attention weight vector, and it completes bidirectional feature interaction and purification through matrix multiplication with the feature map in the other branch as follows:

(6) $$Y_{i}=Sigmoid\left(MatrixMul\left(\sigma \left(Z_{1\times 1}\right),V_{3\times 3}\right)+MatrixMul\left(\sigma \left(Z_{3\times 3}\right),V_{1\times 1}\right)\right)\cdot X_{i}$$

In this interaction formula, σ represents the joint non-linear mapping of 2D global pooling and Softmax activation; *Z* and *V* denote the channel attention weight vector and feature output generated by the corresponding branch, respectively; and $Y_{i}$ is the purified grouped feature.

This multi-scale parallel attention mechanism provides a highly targeted breakthrough solution for the complex background interference in the Pest24 dataset. At the shallow stage, EMA filters out redundant background edges retained and indiscriminately amplified by SPD-Conv. At the deep stage, it further strips away high-dimensional semantic confusions caused by equipment flashlights and agricultural envrionment. From the physical mechanism of feature perception, the parallel 3×3 convolution can precisely lock onto the micro-morphological textures of tiny pests, while the 1×1 branch, equipped with a receptive field spanning the entire image, is able to capture a more global field of view. Through the deep interaction of cross-spatial learning, the network essentially constructs a powerful feature discriminator. It can precisely compare and strip high-frequency environmental noise from effective biological targets in semantic spaces of different depths, forcibly assigning extremely high activation weights to genuine pest regions, thereby fundamentally and substantially suppressing the false positive rate caused by complex agricultural environments.

## 3. Experimental Results and Analysis

### 3.1. Experiment Details Setting

All experiments in this study were conducted on a unified hardware platform to ensure rigorous comparative analysis. The workstation is equipped with an Intel Xeon Gold 6430 processor and a single NVIDIA GeForce RTX 4090 GPU with 24 GB of VRAM. The operating system is Ubuntu, and the deep learning framework is PyTorch 2.0.0 accelerated by CUDA 11.8.

To ensure the reproducibility of our results, the detailed hyperparameter settings are summarized in Table 2. These parameters were initially established based on the default configurations of the YOLO architecture, and they were subsequently fine-tuned to fit the high-resolution inputs and the extremely small targets of the Pest24. During the training phase, to prevent the loss of fine-grained features of extremely small pests, the input images from the Pest24 dataset were resized to a high resolution of 1024×1024 pixels. We employed the AdamW optimizer with a momentum of 0.937 and a weight decay of $5\times 10^{-4}$. The initial learning rate was set to 0.01, and a linear learning rate decay strategy with a final learning rate fraction of 0.01 was applied over the 230 training epochs, preceded by a 3-epoch warmup phase (with a warmup momentum of 0.8 and an initial warmup bias learning rate of 0.1) to stabilize the initial gradient updates. The original dataset was randomly partitioned into a training set (containing 17,764 images), a validation set (5075 images), and an independent test set (2539 images) with a ratio of 7:2:1. Furthermore, to enrich the diversity of the training samples and prevent model overfitting, we employed the default data augmentation techniques inherent to the YOLO framework, such as mosaic with 1.0 probability, random horizontal flipping with 0.5 probability, and HSV color-space adjustments.

### 3.2. Evaluation Metrics

Since object detection tasks involve both classification and localization simultaneously, a single accuracy metric is insufficient to comprehensively evaluate the model’s performance. In this study, Intersection over Union (IoU) is introduced as the core criterion to measure the spatial overlap between the prediction box and the ground truth box. Based on a predefined IoU threshold, we can define positive and negative samples for the detection results and subsequently construct the Precision–Recall (PR) curve. The formula for IoU is as follows:

(7) $$IoU=\frac{Area(\mathrm{Prediction}\cap \mathrm{Ground}\;\mathrm{Truth})}{Area(\mathrm{Prediction}\cup \mathrm{Ground}\;\mathrm{Truth})}.$$

Recall and precision are also essential metrics for evaluating model performance and determining its refinement capability. The formulas for calculating precision and recall are as follows:

(8) $$Precision=\frac{TP}{TP+FP},$$

(9) $$Recall=\frac{TP}{TP+FN}.$$

Here, TP (True Positive) represents the number of positive samples correctly predicted as positive by the model. FP (False Positive) denotes the number of negative samples incorrectly predicted as positive. FN (False Negative) stands for the number of positive samples incorrectly predicted as negative. Precision indicates the proportion of truly correct detections among all samples predicted as positive; whereas Recall (R) represents the proportion of positive samples successfully detected by the model out of all actual ground truth positive samples.

Considering that the Pest24 dataset features extremely small targets and a dense distribution, successfully identifying the existence of a target is our primary priority. Therefore, mAP@50 is adopted as the core evaluation criterion for the model. Here, AP (Average Precision) represents the area under the PR curve for a single category, mAP (mean Average Precision) denotes the average AP across all categories, and mAP@50 specifically refers to the mean average precision calculated under a lenient IoU threshold of 0.5. Their respective formulas are as follows:

(10) $$AP=\int_{0}^{1}P\left(R\right)dR,$$

(11) $$mAP@50=\frac{1}{N}\sum_{i=1}^{N}AP_{i}{|}_{IoU=0.5},$$

where *P* denotes Precision, *R* denotes Recall, and P(R) represents the Precision–Recall curve function. *N* indicates the total number of categories in the dataset (i.e., N=24 for Pest24), and $AP_{i}$ represents the Average Precision for the *i*-th category.

However, relying solely on mAP@50 is insufficient to evaluate the model’s superiority in localization accuracy. Therefore, we also introduce mAP@50:95. The formula for this metric is as follows:

(12) $$mAP@50:95=\frac{1}{10}\sum_{t=0.50}^{0.95}mAP@t,$$

where *t* represents the IoU threshold, which ranges from 0.50 to 0.95 with a step size of 0.05. This metric requires the model to maintain stable performance even under stringent IoU thresholds, aiming to comprehensively assess the precision of the model’s bounding box regression capability.

Meanwhile, to evaluate the computational overhead introduced by the improved model, GFLOPs (Giga Floating-point Operations), model size, latency and the number of parameters are utilized as standard evaluation metrics for model complexity and computational cost.

### 3.3. Comparative Analysis with Other Models

To comprehensively evaluate the performance of the proposed EP-YOLO model in practical agricultural scenarios, we conducted comparative experiments with several mainstream object detection algorithms. In real agricultural Internet of Things systems, edge devices usually have strictly limited computing resources and memory. Therefore, a practical pest detection model must achieve an optimal balance among detection accuracy, parameter count, and inference speed. For this comparison, we selected the classic two-stage detector Faster R-CNN [42], the baseline YOLOv8n, the scaled-up YOLOv8s, and other representative lightweight models across different generations including YOLOv5n, YOLOv10n [43], and YOLOv11n.

The data in Table 3 indicate that traditional heavy models are not suitable for this specific task. Faster R-CNN has the largest parameter size of 41.466 M and a computational cost of 162 GFLOPs, but it only achieves a mAP@50 of 0.605. This shows its poor adaptability to densely distributed and tiny pests. When compared to the scaled-up YOLOv8s, our EP-YOLO achieves the exact same mAP@50 of 0.705 and a slightly higher mAP@50:95 of 0.473. However, EP-YOLO requires less than a third of the parameters and computational cost of YOLOv8s. Furthermore, compared to the baseline YOLOv8n, EP-YOLO improves the mAP@50 and mAP@50:95 by 1.1% and 1.9%, respectively. The addition of the Spatial-to-Depth Convolution (SPD) and Efficient Multi-Scale Attention (EMA) modules only increases the parameter count by a negligible 0.025 M, which proves the efficiency of our structural modifications.

YOLOv10n and YOLOv11n show a noticeable decline in accuracy on the Pest24 dataset, with YOLOv11n dropping its mAP@50 to 0.639. This suggests that the generalized architectural updates in newer models might not be well aligned with the unique challenges of extremely small target detection in complex agricultural backgrounds. In contrast, the targeted design of EP-YOLO successfully addresses these issues. Although the integration of new modules brings a slight increase in inference latency to 9.9 ms, it still operates well within the real-time requirements of typical vision sensors. Considering its model size of just 6.08 MB and a low computational demand of 9.2 GFLOPs, EP-YOLO provides a highly practical solution for deployment on edge computing devices in smart agriculture.

To support these numerical findings, detailed classification cases under complex background interference are provided in Section 3.7, where (a) and (b) represent highly dense and mutual occlusion scenarios, and (c), (d), and (e) represent strong background reflection cases. We focus the visual comparison strictly between EP-YOLO and the baseline model to control variables and isolate the impact of our structural modifications. These specific cases visually demonstrate that the proposed modules effectively suppress severe reflective noise and occlusions, whereas the standard baseline frequently generates false predictions under the same interference.

### 3.4. Ablation Study

#### 3.4.1. Effectiveness of Proposed Modules

Table 4 presents the results of the ablation experiments on the Pest24 dataset. We started with the YOLOv8n baseline, which has a precision of 0.718 and a recall of 0.661. From this starting point, we tested the individual and combined effects of the Spatial-to-Depth Convolution (SPD) and Efficient Multi-Scale Attention (EMA) modules.

Adding only the EMA attention mechanism improves the precision to 0.734 and the overall mAP@50 to 0.700. However, the recall drops significantly to 0.636. This indicates that the EMA module is highly effective at filtering out complex background noise to reduce false detections. But because the baseline feature maps already lose a lot of spatial information during traditional downsampling, the strict filtering of the EMA module also suppresses the remaining weak features of extremely small pests, leading to more missed targets.

On the other hand, replacing the standard convolutions with the SPD module alone actually causes the mAP@50 to drop to 0.685. If we look closely at the other metrics, the recall slightly increases to 0.663 compared to the baseline, but the precision falls to 0.702. The SPD module preserves almost all fine-grained spatial information during the downsampling process. It successfully keeps the tiny pests from disappearing, which explains the higher recall. However, it also brings in a massive amount of background texture noise. Without a proper attention mechanism to focus on the targets, this extra noise severely confuses the network and causes more false positives.

The true value of these two modifications emerges when they are used together in the final EP-YOLO model. With both modules active, the model achieves the highest precision of 0.744 and the highest recall of 0.664, pushing the mAP@50 to 0.705 and the mAP@50:95 to 0.473. They form a very clear complementary relationship. The SPD module provides a rich, uncompressed feature map where the spatial pixels of tiny pests are retained, acting as a reliable information provider. Then, the EMA module acts as a precise filter to clean up the accompanying background noise. This specific combination successfully solves the detection bottleneck for tiny targets. Furthermore, this dual improvement is highly efficient, adding only about 0.025 million parameters and 1.1 GFLOPs to the original baseline.

#### 3.4.2. Structural Exploration

We have further explored the optimal configuration of the integrated modules. We mainly focused on two structural variables as follows: the number of standard convolutions replaced by the Spatial-to-Depth Convolution (SPD) module and the specific placement of the Efficient Multi-Scale Attention (EMA) attention mechanism. The comprehensive experimental results are presented in Table 5.

**Figure 5:**
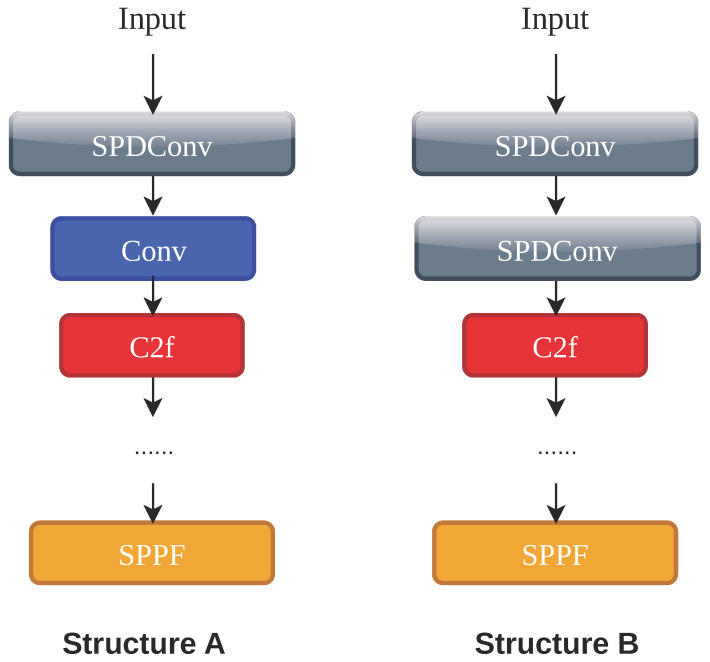
Comparison of network architectures integrating single versus dual SPD modules. (Solid arrows indicate data flow, colors distinguish functional blocks, and ‘…’ denotes structures detailed in Figure 2).

We first looked at the integration number of the SPD module, as visually compared in Figure 5 and detailed in Part A of Table 5. Replacing only the first convolution with a SPD module improves the mAP@50 to 0.699. However, when replacing the first two convolutions with SPD modules, the mAP@50 drops to 0.685. This happens because retaining too much spatial information without a proper filter introduces overwhelming background noise into the network. Moreover, combining just one SPD module with EMA performs poorly, yielding a mAP@50 of 0.682 and causing the precision to drop to 0.656. In contrast, combining two SPD modules with EMA pushes the mAP@50 to a peak of 0.705 and the precision to 0.744. This reveals a strong dependency between the two designs. The EMA filter needs the abundant raw pixel data preserved by the dual SPD structure to work effectively. At the same time, the dual SPD structure relies on the EMA module to clean up the severe background noise it brings in.

**Figure 6:**
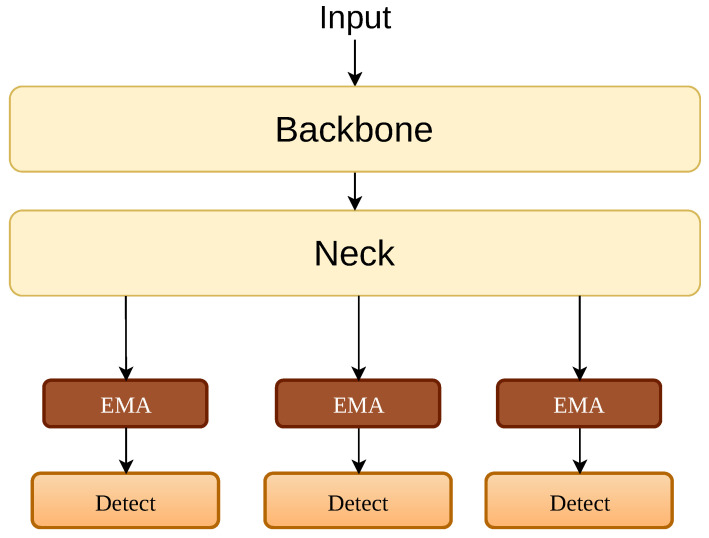
Illustration of placing EMA attention modules in the neck network. (Solid arrows indicate data flow and colors distinguish functional blocks).

Next, we investigated the placement of the EMA modules, as shown in Part B of Table 5. We compared placing three EMA modules in the neck right before the detection heads, which is illustrated in Figure 6, against placing two EMA modules in the backbone. The neck configuration yields a mAP@50 of 0.687, while the backbone configuration achieves 0.700 along with a much higher precision of 0.734. This makes sense from the perspective of feature propagation. If we wait until the neck to apply the attention mechanism, the irrelevant background features have already mixed with the target features during the FPN-PAN process. Filtering the noise early in the backbone intercepts the interference at its source, preventing the noise from spreading to the deeper layers.

**Figure 7:**
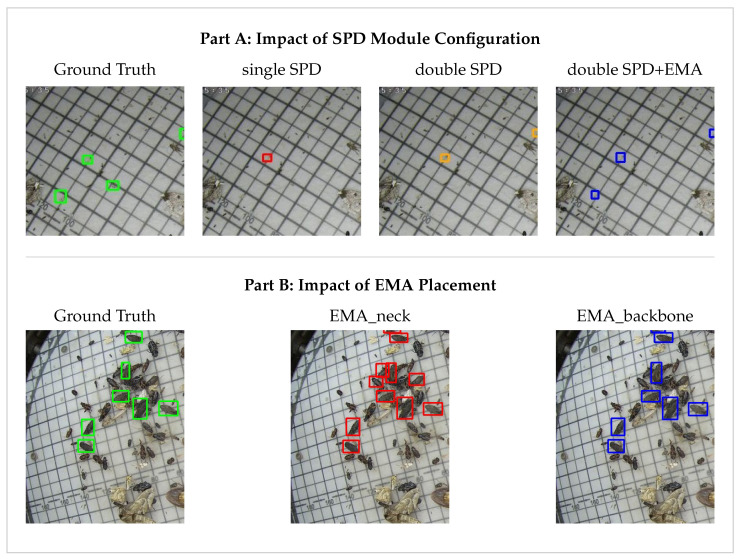
Visual evidence of the Structural Exploration section. Part A demonstrates the impact of SPD Module configuration. Part B demonstrates the impact of EMA placement.

To qualitatively validate these structural choices, Figure 7 provides representative detection results. As shown in Part A, both the single-SPD and double-SPD configurations fail to effectively detect the microscopic targets, whereas the finalized dual SPD combined with EMA outperforms them. Similarly, Part B visually confirms that placing the EMA in the backbone successfully suppresses irrelevant noise, yielding clean and precise detections.

### 3.5. Evaluation of Attention Mechanisms

To verify that the Efficient Multi-Scale Attention (EMA) module is the most suitable attention mechanism for our specific agricultural scenario, we compared it with several classic and lightweight attention modules. We inserted Squeeze-and-Excitation [44], Coordinate Attention [41], a Simple Parameter-Free Attention Module (SimAM) [45], and a Convolutional Block Attention Module (CBAM) [46] into the exact same backbone positions for a fair evaluation.

The results are shown in Table 6. Modules like Squeeze-and-Excitation, CBAM, and the parameter-free SimAM all manage to increase the recall to around 0.68 compared to the baseline, but they cause a severe drop in precision. Squeeze-and-Excitation focuses purely on channel weights and completely ignores the spatial locations where tiny pests are hidden. CBAM includes spatial attention, but its pooling operations tend to blur the features of extremely small targets. SimAM operates without additional parameters, but its energy function is simply not robust enough to handle highly complex leaf and light-trap capture boards backgrounds.

Coordinate Attention performs slightly differently from the others. By capturing positional information, it maintains a relatively high precision of 0.724. However, its overall mAP@50 still drops to 0.689. This indicates that simply knowing the spatial coordinates is insufficient when dealing with pests that vary drastically in scale and shape.

In contrast, EMA is the only module that successfully improves the baseline mAP@50, reaching 0.700. It achieves the highest precision of 0.734 among all tested configurations. While its recall drops to 0.636, this strict filtering behavior is exactly what the model needs to suppress the severe background interference. The success of the EMA module stems from its multi-scale parallel subnetworks and cross-space learning capabilities. It is also worth noting that all tested modules maintain a very similar parameter count of around 3.02 million and require 8.1 to 8.3 GFLOPs. This proves that the superiority of EMA does not come from a larger computational budget.

**Figure 8:**
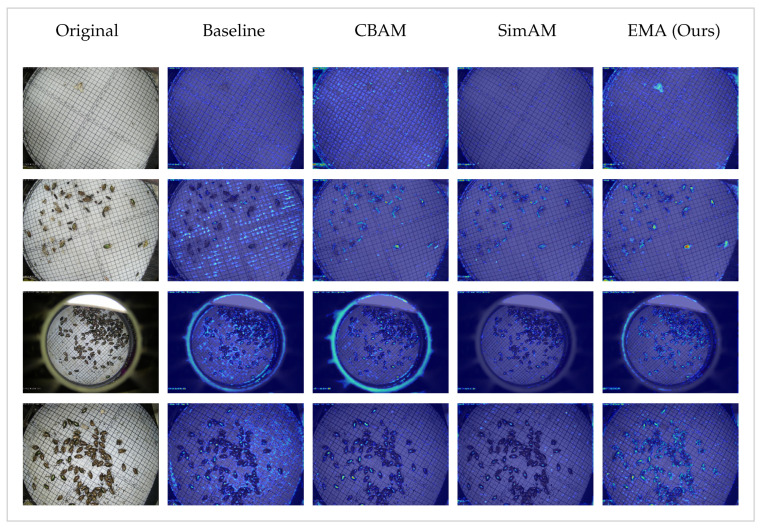
Visual comparison of feature activation maps across different attention mechanisms. (Brighter colors indicate higher significance).

To further support the results we find above, Figure 8 selectively compares the most representative mechanisms using feature activation maps. The visualized samples reveal several distinct behaviors. For the image in Row 1, only EMA successfully localizes the pest, whereas CBAM inadvertently amplifies the background grid noise. Rows 2 and 3 demonstrate responses to complex background and sensor boundary interference. While CBAM and SimAM generally suppress background noise, CBAM mistakenly enhances the circular boundary artifacts (Row 3), and SimAM suffers from degraded target activation. EMA consistently balances this by suppressing boundary noise while maximizing target attention. In Row 4, although CBAM and SimAM clean scattered noise effectively, they partially erode the target’s activation strength. EMA, while retaining slight background sensitivity in this specific case, guarantees robust and sufficient attention on the target itself, preventing the loss of feature.

### 3.6. Performance on Extremely Small and Hard-to-Detect Pests

**Figure 9:**
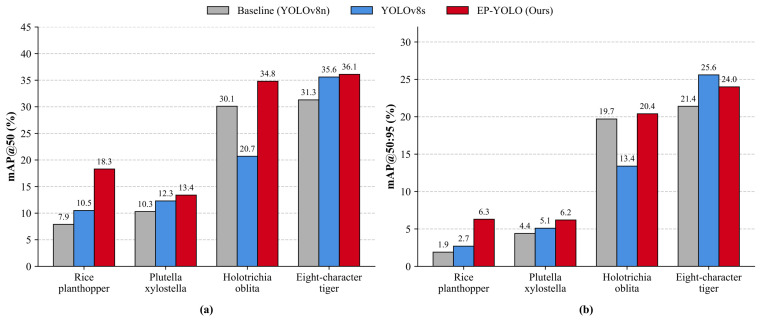
Detection performance comparison on four hard-to-detect pest categories. Part (**a**) shows the mAP@50 results and part (**b**) shows the mAP@50:95 results.

We evaluated the performance of our model on the most difficult tiny pest categories in the Pest24 dataset to see if the structural changes actually work. We compared EP-YOLO with the baseline YOLOv8n and the larger YOLOv8s. Figure 9 shows the results for the following four extremely small categories: *Rice planthopper*, *Plutella xylostella*, *Holotrichia oblita*, and *Eight-character tiger*. Figure 9a shows the mAP@50 comparison. Traditional lightweight models struggle with these targets because of information loss during downsampling. The YOLOv8n baseline only achieves a mAP@50 of 7.9% on the *Rice planthopper*. A common way to handle this problem is to use a larger network. However, using the heavier YOLOv8s provides limited improvement, reaching only 10.5% on the *Rice planthopper*. It even performs worse than the baseline on the *Holotrichia oblita*, dropping from 30.1% to 20.7%. EP-YOLO shows much better results without adding many parameters. It increases the accuracy on the *Rice planthopper* to 18.3%, which is more than double the baseline score. It also outperforms the larger YOLOv8s across all four difficult categories. We can observe similar trends under the stricter mAP@50:95 metric in Figure 9b. This proves that for microscopic targets, adding SPDConv prevents the loss of spatial features, and the Efficient Multi-Scale Attention (EMA) module helps the network focus on the pests. This specific structural design is more effective for tiny targets than just increasing the network size.

**Figure 10:**
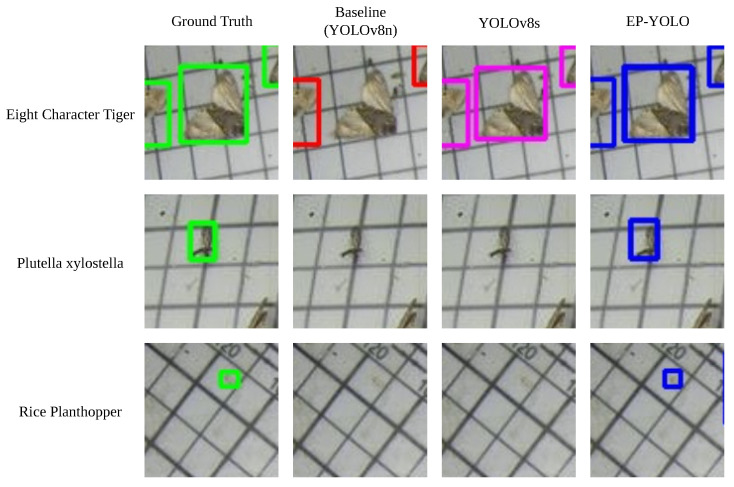
Visual comparison on extremely small pest categories. The rows correspond to the Ground Truth (GT), Baseline, YOLOv8s, and EP-YOLO models. The columns display representative detection results for the *Eight-character tiger*, *Plutella xylostella*, and *Rice planthopper*.

To qualitatively support these numerical findings, Figure 10 presents a visual comparison of detection results across three representative microscopic categories: *Eight-character tiger*, *Plutella xylostella*, and *Rice planthopper*. The matrix demonstrates that while the baseline and YOLOv8s frequently miss these targets due to extreme scale and feature degradation, EP-YOLO successfully localizes them.

### 3.7. Qualitative Visualization Analysis

**Figure 11:**
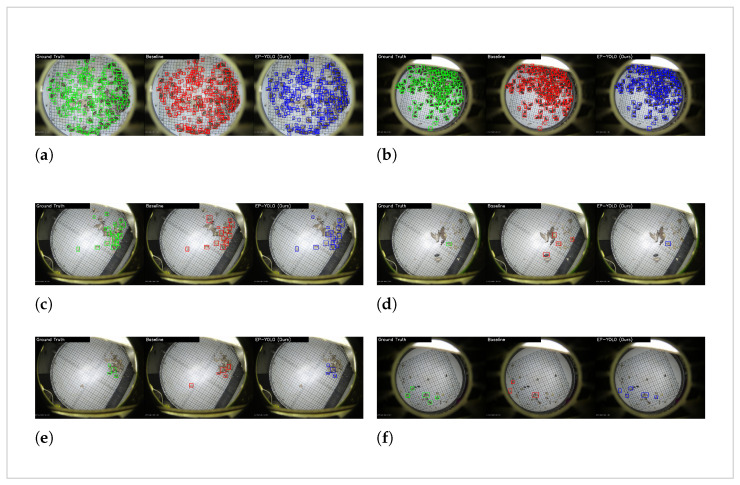
Qualitative detection result comparison. (**a**,**b**) represent highly dense and mutual occlusion scenarios. (**c**–**e**) represent scenarios with strong background reflection. (**f**) represents a scenario with scattered and isolated tiny pests.

We visualized the detection results of EP-YOLO and the baseline model as shown in Figure 11. We selected four typical images from the test set, arranged from (a) to (d). The specific detection counts are recorded in Table 7.

Figure 11a,b show crowded pest trap boards with hundreds of overlapping insects. In (a), the baseline detects 200 targets but makes 53 misdetections. EP-YOLO detects one less target but noticeably reduces the misdetections down to 42. This shows that in highly dense and mutual-occlusions scenes, our model reduces false positive predictions effectively. In (b), EP-YOLO detects one more pest than the baseline and further reduces false detections from 31 to 27, demonstrating better feature distinction when insects heavily overlap.

Figure 11c–e illustrates the detection performance under strong background reflection. In scenario (c), the baseline model is distracted by this noise, missing ten actual pests and making three false predictions. EP-YOLO mitigates this issue, detecting 16 valid targets and reducing the false predictions to zero. Scenarios (d) and (e) further confirm this behavior. The baseline model generates a total of five false predictions across these two images. It also misses one ground truth target in (e). EP-YOLO maintains zero false predictions in both (d) and (e), while successfully detecting all four ground truth targets in (e).

Figure 11d displays a relatively empty board where the few pests are scattered and completely isolated from each other. The baseline model misses four out of the five actual targets. EP-YOLO successfully locates four of them and also keeps a lower false positive count. This indicates that the SPD module preserves the spatial information of isolated tiny targets across the deep layers of the network.

## 4. Discussion

### 4.1. Discussion on Model Architectures and Performance Trade-Offs

As indicated by the comparative results in Table 3, the latest general-purpose architectures, YOLOv10 and YOLOv11, exhibited an unexpected performance degradation on the Pest24 dataset. Specifically, YOLOv10 eliminates the Non-Maximum Suppression (NMS) post-processing to reduce inference latency [43]. However, NMS is a crucial mechanism utilized in previous versions (such as YOLOv8) to filter out redundant bounding boxes in dense areas. Without the NMS mechanism, the model inherently struggles to accurately distinguish individual targets in highly overlapping distributions, directly leading to severe feature aliasing and missed detections in crowded light-trap scenes.

YOLOv11 introduces complex modules such as C3k2 and deep C2PSA spatial attention [47]. However, previous studies indicate that targets occupying merely a few dozen pixels suffer from irreversible spatial resolution collapse during the initial strided downsampling stages [48]. Therefore, the backend enhancements of YOLOv11 fail to address the fundamental issue of early-stage information loss. When the degraded feature maps reach the deep attention modules, the network essentially allocates weights to the amplified background noise rather than the target features. In contrast, EP-YOLO explicitly resolves this issue by utilizing the front-end SPD-Conv to preserve spatial information at the source.

Beyond general baselines, comparing EP-YOLO with recent specialized models further highlights its practical advantages. For instance, Tang et al. [26] proposed an improved Pest-YOLO integrated with a Transformer encoder, achieving a mAP@50 of 73.4%. However, this comes at a prohibitive cost of 84.57 M parameters and 131.7 GFLOPs. A model nearly 28 times larger than EP-YOLO (3.035 M) is highly impractical for edge-computing visual sensors. Moreover, recent lightweight efforts like YOLO-LCE [32] successfully compressed the parameter count to 1.69 M. Yet, this extreme compression caused a severe accuracy drop to 63.9% (6.6% lower than EP-YOLO). Ultimately, our EP-YOLO achieves a mAP@50 of 70.5% with only 3.035 M parameters. By effectively balancing front-end feature retention with the lightweight EMA module, EP-YOLO provides a highly cost-effective and deployable solution for agricultural Internet of Things (IoT) systems.

### 4.2. Limitations and Failure Case Analysis

**Figure 12:**
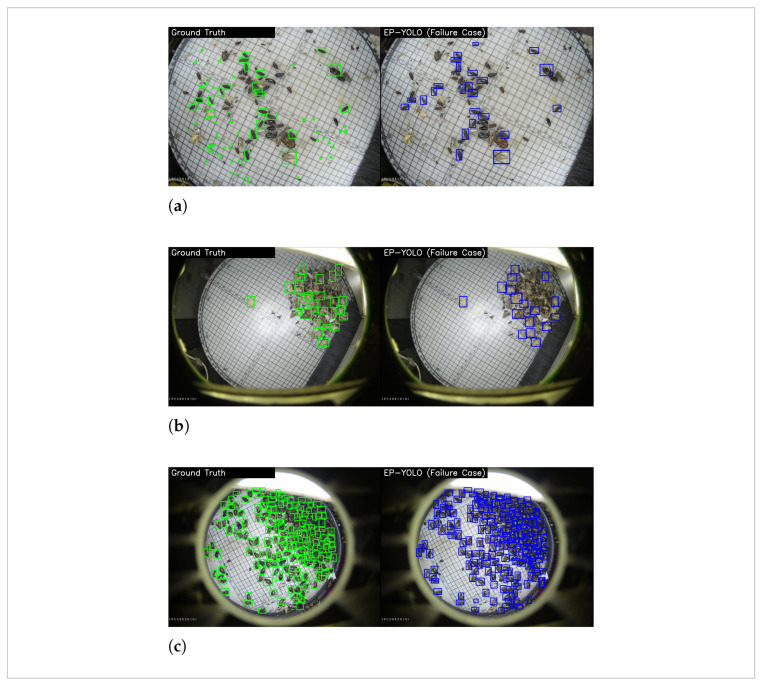
Visualization of typical failure cases for EP-YOLO. (**a**,**b**) represent undetected cases (False Negatives) under extreme scale and overlapping constraints. (**c**) represents misdetected cases (False Positives) in overly crowded scenarios.

To further explore the boundaries of EP-YOLO, we analyzed several typical failure cases. Figure 12 and Table 8 present the visual and quantitative results of these challenging scenarios.

The undetected cases are primarily constrained by the following two physical extremes: absolute small scale and severe overlapping. In Figure 12a, the model failed to detect 66 out of 88 targets, most of which are the smallest pests in dataset, the rice planthopper. This occurs because the physical size of these micro-pests approaches the imaging limit of the camera sensor. The absolute lack of visual features at the pixel level makes detection extremely difficult, even with our front-end spatial preservation strategy. Furthermore, Figure 12b illustrates failures caused by physical overlapping, where 10 out of 26 targets were missed. When pests stack closely on the capture board, their bounding boxes overlap significantly. This dense case inevitably triggers the Non-Maximum Suppression (NMS) mechanism during post-processing, which could filter out valid targets as redundant predictions incorrectly.

Conversely, misdetections are mainly driven by highly dense distributions and complex local textures. As shown in Figure 12c, the model generated 46 false predictions in a crowded region containing 172 ground truth targets. The network easily misinterprets localized noisy textures as independent micro-targets, making the detection head overly sensitive in specific regions. These failure cases indicate that pure two-dimensional object detection faces inherent physical limitations in composite scenarios of extreme scale and high density. Addressing these bottlenecks may require exploring more advanced architectures in future work.

### 4.3. Research Limitations and Future Directions

Although EP-YOLO achieved the highest detection accuracy of 70.5% in comprehensive evaluations, it still faces inherent physical and data-related boundaries in practical agricultural deployment.

The first challenge is the extreme long-tail distribution of the dataset. For instance, *Holotrichia oblita* has only 108 instances in the entire Pest24 dataset. This severe data imbalance makes it exceedingly difficult for data-driven deep models to adequately extract generalized features for minority classes, rendering them highly susceptible to false negatives in real-world scenarios. Future work could introduce dynamic resampling strategies or generative model-based data augmentation techniques for minority classes to mitigate this issue. Secondly, although the model achieved significant relative improvements on extremely small targets—with the mAP of *Rice planthopper* and *Plutella xylostella* increasing to 18.3% and 13.4%, respectively—its absolute accuracy on these categories still remains low. When the target scale approaches the physical limits of optical imaging, relying solely on algorithmic feature enhancement is insufficient to completely overcome the recognition bottleneck. Exploring higher-resolution imaging sensors or multi-modal information fusion represents a potential avenue to break through this ceiling.

Furthermore, the severe aggregation and overlapping of pests on light-trap capture boards exacerbate the detection difficulty for existing paradigms. The similar morphologies of densely packed pests not only trigger visual feature aliasing but also cause bounding boxes generated by the Non-Maximum Suppression (NMS) algorithm to suffer from erroneous suppression or spatial drift in highly occluded regions, thereby compromising counting accuracy. Future research could consider incorporating Density Map Estimation methods or NMS-free, end-to-end detection architectures to tackle this crowding dilemma.

Finally, regarding engineering deployment, although EP-YOLO’s parameter count is strictly constrained to a lightweight standard, theoretically satisfying the computational limits of edge devices, this study has yet to conduct large-scale testing on real-world agricultural devices. Subsequent research will focus on TensorRT quantization acceleration and on-site inference efficiency evaluation of the model on low-power computing platforms.

## 5. Conclusions

In this study, an enhanced lightweight pest detection model named EP-YOLO is proposed. In general, EP-YOLO aims at resolving problems such as micro-pest detection and complex background interference on agricultural light-trap capture boards. We integrated two advanced modules, Spatial-to-Depth Convolution (SPD) and Efficient Multi-Scale Attention (EMA), into our network, which enable EP-YOLO to retain lossless details and precisely suppress background noise. We also designed comprehensive experiments to validate the improvements of EP-YOLO. Evaluations on the Pest24 dataset show that EP-YOLO achieves an excellent mean Average Precision of 70.5%. Performance on extremely tiny pests, such as *Rice planthopper* and *Plutella xylostella*, is also outstanding. While outperforming a series of baseline models, our model strictly aligns with the computational constraints of edge devices for real-time deployment. In conclusion, our study provides a robust visual algorithmic foundation for agricultural pest monitoring, and facilitates future integrations with real-time detection devices to establish early warning systems.

## Figures and Tables

**Table 1:** Description of the 24 categories of pest information from the Pest24 dataset, including the number of images, instances, and scale for each category.

Index	Pest Name	Scale	Number of Images	Number of Instances	Index	Pest Name	Scale	Number of Images	Number of Instances
1	*Rice planthopper*	0.034	316	1511	15	*Spodoptera cabbage*	0.42	1707	2302
2	*Rice Leaf Roller*	0.123	944	1240	16	*Scotogramma trifolii Rottemberg*	0.28	3223	4679
3	*Striped rice borer*	0.186	454	1285	24	*Yellow tiger*	0.398	1388	1686
5	*Armyworm*	0.394	3828	8880	25	*Land tiger*	0.639	369	475
6	*Bollworm*	0.281	9049	28,014	28	*Eight-character tiger*	0.441	154	168
7	*Meadow borer*	0.226	5526	16,516	29	*Holotrichia oblita*	0.334	90	108
8	*Athetis lepigone*	0.13	7520	30,339	31	*Holotrichia parallela*	0.255	3111	11,675
10	*Spodoptera litura*	0.458	1588	1951	32	*Anomala corpulenta*	0.249	5228	53,347
11	*Spodoptera exigua*	0.138	3614	7263	34	*Gryllotalpa orientalis*	0.95	3629	6528
12	*Stem borer*	0.277	1357	1804	35	*Nematode trench*	0.32	118	167
13	*Little Gecko*	0.57	2503	4279	36	*Agriotes fuscicollis Miwa*	0.114	1814	6484
14	*Plutella xylostella*	0.043	531	953	37	*Melahotus*	0.158	239	768

Scale represents the average relative scale (the ratio of the size of annotated bounding box to the size of the original image).

**Table 2:** Hyperparameter settings for the model training phase.

Parameter	Value
Image Size	1024 × 1024
Training Epochs	230
Batch Size	16
Optimizer	AdamW
Initial Learning Rate	0.01
Final LR Fraction	0.01
LR Schedule	Linear
Momentum	0.937
Weight Decay	5 × $10^{-4}$
Warmup Epochs	3
Warmup Momentum	0.8
Warmup Bias LR	0.1

**Table 3:** Performance comparison of EP-YOLO with state-of-the-art object detection models on the Pest24 dataset.

Model	mAP@50	mAP@50:95	Params (M)	Size (MB)	GFLOPs	Latency (ms)
Faster R-CNN	0.605	0.343	41.466	321.0	162.0	20.9
YOLOv5n	0.687	0.438	2.50	5.10	7.1	7.9
YOLOv8n	0.694	0.454	3.01	6.03	8.1	5.4
YOLOv8s	**0.705**	0.462	11.13	21.50	28.5	8.5
YOLOv10n	0.685	0.447	2.27	5.56	6.6	9.7
YOLOv11n	0.639	0.411	2.59	5.28	6.3	10.3
EP-YOLO (Ours)	**0.705**	**0.473**	3.035	6.08	9.2	9.9

Bold values indicate the best results.

**Table 4:** Ablation study on the effectiveness of the proposed SPDConv and EMA modules.

Model	SPD	EMA	mAP@50	mAP@50:95	Precision	Recall	GFLOPs	Params (M)
Baseline (YOLOv8n)			0.694	0.454	0.718	0.661	8.1	3.010
+EMA		✓	0.700	0.456	0.734	0.636	8.3	3.020
+SPD	✓		0.685	0.446	0.702	0.663	9.1	3.025
**EP-YOLO (Ours)**	✓	✓	**0.705**	**0.473**	**0.744**	**0.664**	9.2	3.035

Bold values indicate the best results; the checkmark (✓) indicates the integration of the corresponding module.

**Table 5:** Structural exploration of SPD convolution integration depth and EMA attention placement.

Configuration	mAP@50	mAP@50:95	Precision	Recall	GFLOPs	Params (M)
Part A: Integration number of SPDConv						
1st Conv replaced by SPD	0.699	0.451	0.696	0.657	8.4	3.010
1st & 2nd Convs replaced by SPD	0.685	0.446	0.702	0.663	9.1	3.025
1st Conv by SPD + EMA	0.682	0.449	0.656	0.682	8.5	3.020
1st & 2nd Convs by SPD + EMA (Ours)	**0.705**	**0.473**	**0.744**	0.664	9.2	3.035
Part B: Placement of EMA Attention Mechanism						
EMA inserted in the Neck	0.687	0.445	0.684	**0.686**	8.3	3.020
EMA inserted in the Backbone (Ours)	**0.700**	**0.456**	**0.734**	0.636	8.3	3.020

Bold values indicate the best results.

**Table 6:** Performance comparison of different attention mechanisms integrated into the backbone.

Attention Module	mAP@50	mAP@50:95	Precision	Recall	GFLOPs	Params (M)
Baseline	0.694	0.454	0.718	0.661	**8.1**	**3.010**
SE [44]	0.693	0.454	0.687	0.683	**8.1**	3.020
CBAM [46]	0.689	0.450	0.676	0.679	**8.1**	3.020
CA [41]	0.689	0.452	0.724	0.662	**8.1**	3.020
SimAM [45]	0.687	0.448	0.677	**0.684**	**8.1**	**3.010**
**EMA**	**0.700**	**0.456**	**0.734**	0.636	8.3	3.020

Bold values indicate the best results.

**Table 7:** Qualitative comparison of detection results.

Scenario	Ground Truth	Model	Detected	Undetected (FN)	Misdetected (FP)
(a)	214	Baseline	**200**	**14**	53
		EP-YOLO	199	15	**42**
(b)	150	Baseline	148	2	31
		EP-YOLO	**149**	**1**	**27**
(c)	23	Baseline	13	10	3
		EP-YOLO	**16**	**7**	**0**
(d)	3	Baseline	1	0	3
		EP-YOLO	1	**0**	**0**
(e)	4	Baseline	3	1	2
		EP-YOLO	**4**	**0**	**0**
(f)	5	Baseline	1	4	2
		EP-YOLO	**4**	**1**	**1**

**Table 8:** Quantitative evaluation of EP-YOLO on the selected failure cases.

Scenario	Ground Truth	Detected (TP)	Undetected (FN)	Misdetected (FP)
(a)	88	22	**66**	4
(b)	26	16	**10**	3
(c)	172	164	8	**46**

## Data Availability

The Pest24 dataset analyzed in this study is publicly available. Other data presented in this study are available upon request from the corresponding author.
